# Biomarker Landscape in RASopathies

**DOI:** 10.3390/ijms25168563

**Published:** 2024-08-06

**Authors:** Noemi Ferrito, Juan Báez-Flores, Mario Rodríguez-Martín, Julián Sastre-Rodríguez, Alessio Coppola, María Isidoro-García, Pablo Prieto-Matos, Jesus Lacal

**Affiliations:** 1 Laboratory of Functional Genetics of Rare Diseases, Department of Microbiology and Genetics, University of Salamanca (USAL), 37007 Salamanca, Spain; noemi.ferrito@usal.es (N.F.); alumni.jbaez@usal.es (J.B.-F.); juliansastre@usal.es (J.S.-R.); acoppola@usal.es (A.C.); 2 GIR of Biomedicine of Rare Diseases, University of Salamanca (USAL), 37007 Salamanca, Spain; pprieto@saludcastillayleon.es; 3 Institute of Biomedical Research of Salamanca (IBSAL), 37007 Salamanca, Spain; misidoro@saludcastillayleon.es; 4 Clinical Biochemistry Department, University Hospital of Salamanca, 37007 Salamanca, Spain; 5 Clinical Rare Diseases Reference Unit DiERCyL, 37007 Castilla y León, Spain; 6 Department of Medicine, University of Salamanca (USAL), 37007 Salamanca, Spain; 7 Department of Pediatrics, University Hospital of Salamanca, 37007 Salamanca, Spain; 8 Department of Biomedical and Diagnostics Science, University of Salamanca (USAL), 37007 Salamanca, Spain

**Keywords:** RASopathies, biomarkers, molecular signatures, precision medicine, personalized diagnostics

## Abstract

RASopathies are a group of related genetic disorders caused by mutations in genes within the RAS/MAPK signaling pathway. This pathway is crucial for cell division, growth, and differentiation, and its disruption can lead to a variety of developmental and health issues. RASopathies present diverse clinical features and pose significant diagnostic and therapeutic challenges. Studying the landscape of biomarkers in RASopathies has the potential to improve both clinical practices and the understanding of these disorders. This review provides an overview of recent discoveries in RASopathy molecular profiling, which extend beyond traditional gene mutation analysis. mRNAs, non-coding RNAs, protein expression patterns, and post-translational modifications characteristic of RASopathy patients within pivotal signaling pathways such as the RAS/MAPK, PI3K/AKT/mTOR, and Rho/ROCK/LIMK2/cofilin pathways are summarized. Additionally, the field of metabolomics holds potential for uncovering metabolic signatures associated with specific RASopathies, which are crucial for developing precision medicine. Beyond molecular markers, we also examine the role of histological characteristics and non-invasive physiological assessments in identifying potential biomarkers, as they provide evidence of the disease’s effects on various systems. Here, we synthesize key findings and illuminate promising avenues for future research in RASopathy biomarker discovery, underscoring rigorous validation and clinical translation.

## 1. Introduction

RASopathies represent a group of rare genetic diseases affecting fewer than 1 in 1000 people worldwide, characterized by a germline mutation in one of the genes encoding components of the RAS/MAPK pathway [1,2]. These mutations may be de novo or inherited, and the associated phenotypic features and congenital anomalies are diverse [3]. These conditions share overlapping features, including developmental delays, craniofacial dimorphism, cardiac malformations, cutaneous manifestations, musculoskeletal abnormalities, neurologic issues, and an elevated risk of cancer, making patients’ lifespan shorter [3]. These diverse and often severe symptoms significantly affect patients’ quality of life, impacting multiple organ systems. For this reason, it is important to advance research in this area to improve diagnosis, treatment, and support for affected individuals. The spectrum of RASopathies encompasses diverse disorders such as neurofibromatosis type 1 (NF1), Noonan syndrome (NS), Noonan syndrome with multiple lentigines (NSML), Noonan syndrome-like with loose anagen hair (NS-LAH), neurofibromatosis–Noonan syndrome (NFNS), Noonan syndrome-like (NSL), Legius syndrome (LS), cardiofaciocutaneous syndrome (CFC), Costello syndrome (CS), and capillary malformation–arteriovenous malformation syndrome (CM-AVM) [4]. NF1 results from various types of loss-of-function mutations in the tumor suppressor gene *NF1*. NS presents the highest locus heterogeneity: approximately half of NS patients carry mutations in *PTPN11*, while the other half present mutations in *SOS1*, *SOS2*, *SPRY1*, *CBL*, *LZTR1*, *RIT1*, *RRAS2*, *NRAS*, *HRAS*, *RRAS*, *KRAS*, *MRAS*, *PPP1CB*, *CRAF*, *RAF1*, *MAP3K8*, and *ERK2* [3,5]. While NS arises from gain-of-function mutations in RAS pathway proteins, NSML is due to loss-of-function mutations [6]. NS-LAH arises from mutations in *SHOC2* or *PPP1CB* [7,8], whereas NFNS arises from mutations in *NF1* and may involve the co-occurrence of variants in *NF1* and another gene associated with NS [9]. LS is caused by mutations in *SPRED1* [10]; CFC results from mutations in *KRAS*, *BRAF*, *MEK1*, and *MEK2* [11]; CS is caused by mutations in *HRAS* [12]; and CM-AVM is most frequently caused by mutations in *RASA1*, although recently mutations in *EPHB4* have been identified [13]. RASopathy patients exhibit alterations in the expression and activation of proteins in several signaling pathways, including RAS/MAPK, PI3K/AKT/mTOR, Rho/ROCK/LIMK2/cofilin, cAMP/PKA, JAK/STAT, Hippo, Wnt/β-catenin, and TGF-β [14,15,16,17]. Given the overlapping clinical presentations among RASopathies alongside their unique characteristics, leveraging biomarkers becomes imperative to enhance diagnostic accuracy, prognostic assessment, and treatment strategies. Moreover, there is a need for continued exploration and validation of these biomarkers to better understand their roles and potential clinical applications.

A biomarker is a measurable and quantifiable indicator of normal or pathological processes or a response to exposures or interventions. Biomarkers can be divided into different categories based on the evaluated indicator: molecular (DNA, RNA, proteins, and metabolites), histologic (tissue samples), or physiologic (blood pressure, heart rate, electrocardiography, echocardiography, and energy X-ray absorptiometry). An ideal biomarker should be either binary (absent or present) or objectively quantifiable, and it should be sensitive and specific. For this reason, developing a biomarker is a lengthy process that requires several steps, including identification, validation, and characterization [18]. Depending on their specific use, biomarkers can be categorized into diagnostic, monitoring, pharmacodynamic/response, and prognostic categories, and for their different uses, they assume great biological relevance [19]. A diagnostic biomarker identifies and confirms the presence and subtype of disease, playing a pivotal role in redefining disease classification in the precision medicine era. Early disease diagnosis enables earlier patient treatment, making the design of early diagnostic biomarkers crucial. Additionally, a prognostic biomarker predicts the likelihood of clinical events, disease recurrence, or progression and is essential for assessing the risk of poor outcomes. Monitoring biomarkers are indispensable for tracking disease progression, as they reflect disease status and exposure to or effects of medical agents. They also aid in treatment tailoring by providing evidence of off-target effects and ensuring drug safety through stable biomarker levels [19]. Furthermore, biomarkers enable an understanding of the molecular basis of physiological or pathological mechanisms, and they can also be essential in drug development and personalized medicine, as they make it possible to tailor treatments to the molecular profiles of patients [20].

Historically, NF1 was the first described RASopathy, diagnosed through clinical analysis and characteristic phenotypical features such as café-au-lait spots, intertriginous freckling, neurofibromas, and skeletal dysplasia, among other physiological and histological biomarkers [2]. The development of genetic testing techniques in the 2000s enabled precise identification of various mutations, leading to more accurate diagnoses despite overlapping characteristics. Today, the advent of next-generation sequencing (NGS) technologies allows a deeper analysis of variants causing RASopathies [20]. Currently, biomarkers used in RASopathies include genetic and transcriptional analysis of the RASopathy-associated genes, activation levels of RAS/MAPK proteins, measurement of serum levels of immune markers involved in the RAS/MAPK pathway, cardiac biomarkers, neurocognitive assessments, and physical features [3,21,22,23,24]. Nonetheless, only a few biomarkers are used in the detection and monitoring of RASopathies, and furthermore, there is a lack of specific therapies.

Even today, a significant number of RASopathy patients test positive in clinical analysis but not in genetic testing, due to the presence of mutations in non-coding regions or mosaicism [25]. Furthermore, despite the extensive literature on RASopathies and their molecular signatures, very few of these findings are employed as biomarkers in clinical practice, not only for diagnosis but also for tailoring precision medicine. The validation of a new biomarker for clinical use is a large, multi-step process ensuring its reliability, accuracy, and utility, involving analytical validation to establish its characteristics and biological relevance, followed by clinical validation and utility through retrospective and prospective clinical trials to confirm its ability to predict clinical outcomes reliably [18]. For RASopathies, the primary challenge for a biomarker is achieving high sensitivity and specificity in distinguishing between closely related disorders reproducibly, especially given the absence of RASopathy-specific treatments and the need for drugs targeting comorbidities or specific pathological features, necessitating a wide array of biomarkers [2]. The aim of this review is to consolidate existing knowledge on biomarkers utilized in the diagnosis, monitoring, and treatment of RASopathies, while also proposing avenues for future research to identify novel biomarkers. To achieve this goal, we have summarized recent studies and findings on RASopathies, categorizing biomarkers into molecular, metabolic, histological, and physiological sections.

## 2. Molecular Biomarkers of RASopathies

Molecular biomarkers are defined as one or a group of individual molecules measured by differential expression or concentration between a disease state and a normal control [26]. Molecular RASopathy biomarkers used thus far include genetic variants evaluated by genetic testing, mRNAs and ncRNAs assessed through both qualitative and quantitative analysis, and protein activity and expression levels [26].

### 2.1. Genes as Biomarkers

**Figure 1:**
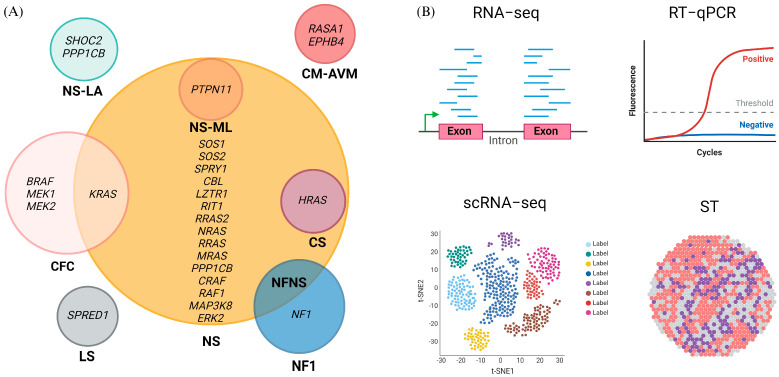
Molecular biomarkers of RASopathies. (**A**) Each circle represents a specific RASopathy and includes the associated genes. (**B**) mRNA detection methods: Overview of the main mRNA detection methods used to study the RASopathies. Created with BioRender.com (accessed on 3 August 2024).

The most employed method of diagnosis of RASopathies is clinical examination; nonetheless, genetic testing is the most reliable method of detection and a well-established source of diagnostic molecular biomarkers, even if it is not routinely conducted for all patients [26]. This includes genomic DNA analysis of the RASopathy genes (Figure 1A) to find the pathogenic genetic variants by Sanger sequencing as well as whole-genome sequencing (WGS) and whole-exome sequencing (WES) for more comprehensive genetic testing [22,27]. Identified variants are normally classified following the criteria of the Human Genome Variation Society as benign, likely benign, uncertain clinical significance, likely pathogenic, or pathogenic, so that they can be used as genetic biomarkers of the disease [28,29]. Nonetheless, genetic screenings may sometimes prove ineffective in detecting the disease, as seen in the case of NS, where approximately 10–20% tested negative [25]. Furthermore, mosaicism, a condition in which individuals have two or more populations of cells with different genotypes due to spontaneous new mutations occurring during early embryonic or fetal development, can also contribute to the challenges in diagnosing RASopathies, as detection in blood is rarely successful [30]. In such cases, it is necessary to directly test the affected tissue by somatic sequencing to identify the mutation [31].

### 2.2. mRNA Biomarkers

The second most common type of biomarker used in RASopathies is at the RNA level, involving qualitative and quantitative analysis of mRNA and non-coding RNA (ncRNA), such as microRNAs (miRNAs) and long non-coding RNAs (lncRNAs) [26,32]. In various studies, mRNA has been utilized as a diagnostic and prognostic biomarker and as a tool to uncover the molecular mechanisms underlying RASopathies, employing diverse transcriptomics techniques (Figure 1B, Table S1) [32,33,34,35].

In NF1, certain pathogenic variants can impact pre-mRNA splicing, and they are typically missed by genetic testing and bioinformatic prediction. For this reason, mRNA analysis has been crucial for identifying these variants and diagnosing the disease [33,34]. mRNA has also been used as a prognostic biomarker in CS. RT-qPCR studies demonstrated that the severity of the phenotype and the frequency of cancer in this syndrome may result from the splicing efficiency of exon 2 inclusion due to activating HRAS mutations. This also suggests that therapeutic interventions for CS could potentially target exon 2 skipping [35]. Furthermore, mRNA studies enable the observation of differential gene expression in tissues and cell types, potentially revealing gene expression profiles of both affected and unaffected tissues in RASopathies. Although these differences have not yet been used as biomarkers of RASopathies, they could be crucial for designing new biomarkers in the future (Table S2) [26,36]. In NS, RNA-seq profiles of WT and *LZTR1*-deficient induced pluripotent stem cell-derived cardiomyocytes (iPSC-CMs) have already been analyzed in a preclinical model of CRISPR repair for NS-associated cardiomyopathy, demonstrating that such therapy could potentially offer a means to normalize the hypertrophic phenotype commonly observed in NS patients [37].

Gene expression analysis in cardiomyocytes from an NSML patient revealed more than 400 downregulated genes and 200 upregulated genes, with the latter being genes associated with muscle development [38]. Furthermore, RNA-seq analysis has been utilized in CM-AVM to assess whether genetic variants causing the disease could result in a reduction in *RASA1* or RAS/MAPK gene expression [39]. Regarding NF1, it was discovered that the extracellular matrix of cutaneous neurofibromas (cNFs) in NF1 patients is largely comprised of collagen VI, a collagen type associated with a pro-tumorigenic role, in contrast to the typically pro-fibrogenic collagen I [40]. Furthermore, a study analyzing mRNA profiles of the plexiform neurofibroma (pNF) tumor environment demonstrated that these tumors are enriched with fibroblasts and non-myelinated Schwann cells [32]. Moreover, through the analysis of differential gene expression using scRNA-seq, it was revealed that orbitofacial NFs exhibit heightened activation of pathways related to cell proliferation, interferons, and immunity, suggesting a potential explanation for their increased local aggressiveness compared to other types of NFs [41]. All this evidence highlights the potential of incorporating mRNA into clinical practice and diagnosis.

### 2.3. ncRNA Biomarkers

The majority of the human genome is transcribed into non-coding RNAs (ncRNAs), which can be divided into classes according to their function, shape, and length [42]. Among these, two important categories found to have an impact on RASopathies are microRNAs (miRNAs) and long non-coding RNAs (lncRNAs) [43,44]. Indeed, up to 49 ncRNAs in total have been found to be involved in RASopathies (Table S3). Nonetheless, these are not yet widely used in clinical practice for diagnosing or monitoring RASopathies.

miRNAs serve as fine-tuning negative regulators of gene expression and, when mutated, can lead to RASopathies [43]. A study involving five patients with previously diagnosed RASopathies identified novel variants in mature miRNAs, pinpointing these as the underlying causes of the diseases. Therefore, both genomics and transcriptomics can be employed to analyze miRNAs and discover new variants associated with RASopathies, which are typically not identified through gene sequencing [43]. Furthermore, differential expression of miRNAs can be observed in RASopathies and has been crucial for understanding the molecular signature of malignant peripheral nerve sheath tumors (MPNSTs) compared to pNFs, as well as high-grade gliomas versus low-grade gliomas in NF1 [45,46,47]. Similarly, it has been valuable in clarifying the progression of juvenile myelomonocytic leukemia (JMML) in patients with NS caused by mutations in *PTPN11* [48]. These promising findings highlight the important role of miRNAs in RASopathies and their potential as biomarkers, underscoring the need for further experimental research.

Less is known about lncRNAs; they interact with proteins and nucleic acids to regulate gene expression, yet research into their role in RASopathies remains limited [49]. The lncRNA ANRIL is the only currently reported NF1 lncRNA, and the polymorphism rs2151280 has been associated with pNFs in NF1 patients. Interestingly, this polymorphism has also been identified in optic gliomas in NF1 patients, and some tumors from NF1 patients have exhibited loss of heterozygosity (LOH) at the ANRIL locus [50]. In another study, it was used as a diagnostic biomarker: a boy who was clinically diagnosed with NS but had negative genetic testing was found to have a deletion in lncRNA-*Dnm3os*, as determined by oligonucleotide-based array comparative genomic hybridization [44]. This lncRNA, which encodes two *N-Ras*-regulating miRNAs, was demonstrated to be required for maintaining the proliferative potential of articular chondrocytes by triggering Nerve Growth Factor (NGF) signaling [44]. This evidence not only resolved a particular case of NS but also highlighted a new class of lncRNAs, which, apart from producing miRNAs, form a regulatory network that maintains a proper pool of proliferating chondrocytes to support bone growth, dysregulated in NS.

### 2.4. Protein Biomarkers

**Figure 2:**
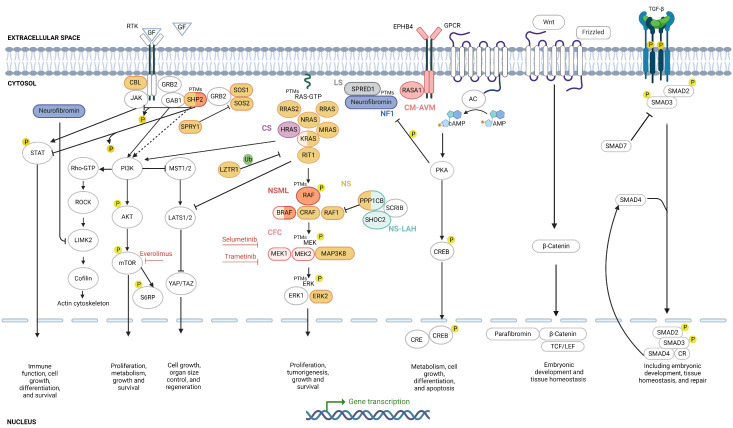
Protein biomarkers of RASopathies. Each color represents a disease: NF1 (blue), LS (gray), CM-AVM (pink), NS (brown), CS (violet), NSML (orange), and CFC (light pink). Inhibitors are indicated in red. Yellow circles denote phosphorylation, and green circles denote ubiquitination. PTM indicates the presence of multiple types of posttranslational modifications on that protein. Created with BioRender.com (accessed on 3 August 2024).

As RASopathies result from specific genetic mutations that can alter protein expression or activation levels directly or indirectly, the use of proteins as biomarkers is crucial in these diseases. To date, 20 proteins have been utilized as biomarkers in RASopathies, with posttranslational modifications (PTMs) including sixteen phosphorylations, two dephoshorylations, five ubiquitinations, two SUMOylations, three methylations, two acetylations, one palmitoylation, one ADP-ribosylation, one O-GlcNAcylation, and one S-nitrosylation being analyzed (Figure 2, Table S4).

#### 2.4.1. RAS/MAPK Biomarkers

The RAS/MAPK pathway biomarkers, including HRAS, KRAS, NRAS, ERK, MEK, RAF, and AKT, are common biomarkers [51] (Figure 2). This pathway is also intricately regulated by PTMs such as phosphorylation, ubiquitination, SUMOylation, and methylation (Figure 2, Table S4) [52]. These modifications are crucial for precise control of RAS signaling, and their dysregulation can contribute to RASopathies and tumorigenesis. RAS protein levels are regulated by ubiquitination and SUMOylation, which play a crucial role in regulating this pathway [53,54]. Additional PTMs regulating RAS degradation include phosphorylation of HRAS by GSK3β on residues T144 and T148 [55]. Other PTMs include methylation of K5 and K147 of KRAS [56] and palmitoylation of C181 and C184 of HRAS and NRAS, respectively, affecting the subcellular location and activity of RAS [57]. PTMs, facilitated by various proteins, play a fundamental role in regulating the degradation of RAS proteins, ensuring tight control cover their signaling functions. The interplay of these PTMs is vital for proper RAS signaling, and disruptions in these modifications can lead to sustained RAS activation, contributing to RASopathies and tumorigenesis. Understanding these PTMs provides valuable insights into the molecular mechanisms underlying RAS-driven diseases and highlights potential therapeutic targets for intervention.

Beyond RAS proteins, neurofibromin levels have been studied as biomarkers in NF1 [58,59,60]. Additionally, mutants such as KRAS^G13D^ exhibit impaired NF1 binding and are specific biomarkers for predicting sensitivity to EGFR-targeted therapies [61]. Interestingly, neurofibromin also undergoes phosphorylation at site S2808 by PKC-ε, which is a crucial regulatory process that impacts the protein’s function in nuclear import and chromosome congression during mitosis [62]. Neurofibromin has long been implicated using various cell types and animal models as a positive or negative regulator of cAMP levels, although identifying the key molecules involved in this coupling has proven challenging, and the role of Ras has been reported to be variable [62]. Additionally, neurofibromin responds to growth factor stimuli, which modulate its interaction with regulatory proteins such as 14-3-3, thereby affecting its GAP activity towards Ras [63]. In NF1, the loss of neurofibromin results in defective regulation of RAS ubiquitination, leading to sustained RAS activation and contributing to tumorigenesis [58]. Regarding PTMs, neurofibromin undergoes methylation [64] and SUMOylation, with 15 SUMO consensus motifs and two SIM sites predicted by JASSA [65]. Palmitoylation also occurs in the SPRED1–neurofibromin–KRAS complex [66]. Other proteins with PTMs include BRAF, c-RAF (RAF1), MEK1, MEK2, and ERK [53,54,55,56,67,68,69,70,71,72,73,74,75,76,77] (Table S2).

#### 2.4.2. PI3K/AKT/mTOR Biomarkers

Due to the crosstalk between the PI3K/AKT/mTOR and RAS/MAPK pathways, phosphorylation levels of AKT, S6RP, and mTOR have been previously used as biomarkers in research on RASopathies [16,78] (Figure 2). To the best of our knowledge, these are not currently used as diagnostic biomarkers for RASopathies. Nonetheless, based on the following evidence, we propose their use in clinical practice for the study of affected tissues. In a case study of a patient with *PTPN11*-caused NS, immunohistochemistry of a lesion from a glioneuronal neoplasm showed positive staining for pmTOR [16]. Similar results were obtained in an NSML patient with hypertrophic cardiomyopathy caused by a mutation in *PTPN11*, where the levels of pAKT and pS6RP (a downstream target of mTOR) in the patient’s skin fibroblasts demonstrated enhanced PI3K/AKT/mTOR pathway activity. Furthermore, treatment with everolimus, an mTOR inhibitor, resulted in an improvement in heart failure risk and a reduction in brain natriuretic peptide levels [78]. Nonetheless, the functional link between SHP2 (encoded by *PTPN11*) and mTOR is not fully understood, although it has been found that the regulatory subunit p85 of PI3K interacts directly with SHP2 [79]. High levels of pAKT, pmTOR, and pS6RP have also been found in MPNSTs, indicating their role in the aggressive clinical behavior of these tumors in NF1 patients [80]. Preclinical and clinical trials have demonstrated the effectiveness of mTOR inhibitors in treating cNFs and low-grade gliomas, underscoring the potential of pmTOR as a treatment response biomarker for these conditions in NF1 patients [81,82,83]. Furthermore, it would be valuable to investigate whether pmTOR could serve as a predictive biomarker for progression from benign to malignant fibromas in NF1.

#### 2.4.3. Rho/ROCK/LIMK2/Cofilin Biomarkers

The Rho/ROCK/LIMK2/cofilin pathway is a key pathway for various cellular processes such as cytoskeletal dynamics, cell migration, cell morphology, and cell adhesion [84] (Figure 2). This pathway has not been extensively studied in the context of RASopathies [85,86], but in NF1, ROCK plays a crucial role in regulating actin cytoskeleton dynamics and cell contractility [85]. Dysregulation of this pathway can lead to cardiac abnormalities and developmental defects, as seen in conditions such as NS and LS, due to gain-of-function SHP-2 mutations that induce hyperactivity of ROCK [87].

#### 2.4.4. cAMP/PKA Biomarkers

Up to three biomarkers have been identified in the cAMP/PKA pathway in RASopathies, namely, cAMP [88,89], PKA [60], and the ratio between neurofibromin isoforms I and II [90]. These molecules influence the balance between the cAMP/PKA and RAS/MAPK pathways, which can be used as a therapeutic strategy for cNFs [89] (Figure 2). The cysteine-serine-rich domain of neurofibromin may regulate adenylate cyclase (AC) activity; thus, the deletion of neurofibromin results in dysfunctional cAMP signaling [88,91]. On the other hand, PKA phosphorylates neurofibromin, impairing its GAP activity and thus dysregulating the RAS/MAPK pathway [60]. The ratio between neurofibromin isoforms I and II contributes to this regulation, as isoform II is a weaker negative regulator of RAS than isoform I [90].

#### 2.4.5. JAK/STAT Biomarkers

The JAK/STAT pathway is composed of non-receptor tyrosine protein kinases (JAKs) and signal transducers and activators of transcription (STATs) [92]. This pathway plays integral roles in various cellular processes, including mitosis, differentiation, apoptosis, hematopoiesis, the development of the immune system, and the functioning of exocrine glands [93]. In RASopathies, this pathway’s components have not yet been used as biomarkers. Given the interplay between the JAK/STAT and RAS/MAPK pathways [94] (Figure 2), it was suggested that this pathway should be explored as a biomarker for RASopathies. Indeed, SHP2 directly dephosphorylates STAT5 and JAK1, thereby attenuating their activity through inhibition of dimerization [95,96]. Additionally, STAT3 is identified as a critical factor in the initiation of neurofibromas [97].

#### 2.4.6. Hippo Pathway Biomarkers

The Hippo tumor suppressor pathway is an evolutionarily conserved signaling cascade that regulates numerous biological processes, such as cell growth, organ size control, and regeneration [98,99] (Figure 2). Although, to the best of our knowledge, the components of the Hippo pathway have not yet been used as diagnostic or monitoring biomarkers in RASopathies, there is evidence highlighting its impact in NF1. WES studies in NF1 patients assessing the role of acquired somatic mutations in the growth of cutaneous neurofibromas (cNFs) have revealed significant dysregulation of Hippo signaling, suggesting its involvement in cancer progression [100]. Further investigations using WES datasets of cNFs from NF1 patients identified 30 somatic mutations in the Hippo pathway genes, along with elevated expression levels of YAP and TAZ [15]. Moreover, evidence suggests that the transformation of MPNSTs from SCs is influenced by deregulation of the Hippo pathway [68,101]. These studies, along with the interplay between the Hippo pathway and the RAS/MAPK and PI3K/AKT/mTOR pathways mediated by core Hippo kinase proteins (MST1/2 and LATS1/2) [14], suggest that investigating Hippo pathway components as biomarkers could yield novel diagnostic and monitoring tools for RASopathies, improving disease management and treatment outcomes.

#### 2.4.7. Wnt/β-Catenin Biomarkers

The Wnt/β-catenin pathway, the canonical pathway involving the Wnt cascade [102], plays crucial roles in embryonic development and tissue homeostasis [103]. The key molecule in this pathway is β-catenin, which acts as a nuclear effector of the pathway and is also an important component of the cytoskeleton [104]. Up to four biomarkers have been related to the Wnt/β-catenin pathway in RASopathies, namely, β-catenin [17,105], the Wnt ligand Frizzled (FZD) [17], neurofibromin [17], and parafibromin [106] (Figure 2). These biomarkers collectively provide insights into the interplay between the Wnt/β-catenin and RAS/MAPK pathways, crucial for understanding the molecular mechanisms underlying RASopathies.

#### 2.4.8. TGF-β Pathway Biomarkers

Transforming growth factor-β (TGF-β) represents an evolutionary conserved family of secreted proteins with cell-type-specific and developmental-stage-specific actions, playing roles in embryogenesis, differentiation of most cell lineages, and adult tissue homeostasis [107,108]. Three biomarkers have been identified for the TGF-β pathway in RASopathies (Figure 2). TGF-β itself is the primary biomarker, as the frequency of CD4+ cells expressing it is increased in NF1 patients, suggesting an immunosuppressed status [109]. SHP2 is a biomarker for NS, since mutations excessively activate the TGF-β pathway, producing an impairment in early neuroectodermal development in NS-iPSCs [110]. Finally, regarding CS, hyperactivation of SMAD3 signaling during osteogenic differentiation of CS-patient-derived mesenchymal stem cells leads to aberrant expression of extracellular matrix remodeling proteins [73]. Understanding the TGF-β signaling pathway might clarify the molecular pathogenesis of tumor development in NF1 and how neurodevelopment and osteogenic differentiation are affected in NS and CS, respectively. The integration of molecular biomarkers with clinical data, including blood tests, cardiac and immune biomarkers, and imaging techniques such as MRI and PET, enhances diagnosis, monitoring, and treatment tailoring for patients. This approach is crucial in clinical practice, providing a more holistic understanding of RASopathies.

## 3. Metabolite Biomarkers

Studying the metabolome can reveal the organism’s metabolic response to a pathological stimulus and provide information on the molecular pathways involved in the development and progression of a certain disease, helping to bridge the genotype–phenotype gap [111,112]. Very few studies have been conducted to correlate metabolomics and RASopathies, and only a limited number of relevant metabolites have been identified. Metabolomic studies on RASopathies have identified up to five different potential biomarkers: adipokines, glucose levels, high-density lipoprotein (HDL) cholesterol, triglycerides (TGs), and urinary catecholamine. It has been demonstrated that individuals with NF1 exhibit increased metabolic levels of adipokines—cytokines regulated by adipocytes—compared to controls [113]. NF1 patients also exhibit reduced fasting blood glucose and enhanced glucose clearance compared to matched controls [113,114]. This enhanced metabolism of glucose and other energy substrates may participate in tumor growth and transformation [115]. Furthermore, other research suggests that both children and young adults affected by NS or Noonan-related diseases have an unfavorable metabolic profile with low HDL cholesterol, a tendency toward elevated TGs, and an impairment in glucose metabolism despite presenting a lean phenotype [116]. Elevated urinary catecholamine metabolites were observed in a patient suffering from progressive neonatal hypertrophic cardiomyopathy (HCM) and dysmorphic features in whom a well-known NSML-associated *PTPN11* mutation (c.1403 C>T; p.T468M) and a novel, potentially pathogenic missense *SOS1* variant (c.1018 C>T; p.P340S) were found. In particular, mild elevations of vanillylmandelic acid (VMA) and homovanillic acid (HVA) raised concerns of neuroblastoma that, along with the progressive HCM, suggested a RASopathy [117]. This altered metabolic profile has also been described in patients with CS, with increased levels of VMA, HVA, epinephrine, norepinephrine, dopamine, and other metabolites [118]. The detection of such metabolites is feasible in terms of accessibility, as the measurements are taken from urine or blood, for example, and the alterations are determined with precision by using methods such as analytical liquid chromatography–mass spectrometry (LC-MS) [119]. Alterations in glucose metabolism and lipid profiles significantly impact disease progression in RASopathies. Increased glucose clearance can support tumor development and exacerbate disease symptoms, while abnormal lipid profiles, such as low HDL cholesterol and high triglycerides, contribute to cardiovascular issues and metabolic complications. These metabolic disruptions fuel tumor growth, elevate cardiovascular risks, and disrupt overall metabolic balance, complicating patient management and outcomes.

## 4. Biomarkers in Histology and Molecular Tissue Characterization

**Figure 3:**
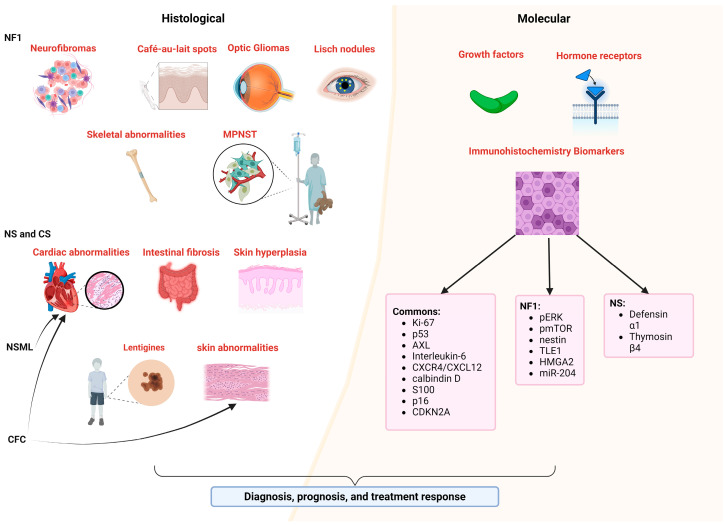
Histological and molecular characterization of RASopathies. Schematic representation of histological (**left panel**) and molecular (**right panel**) biomarkers commonly used in RASopathies for diagnosis, prognosis, and treatment response. Created with BioRender.com (accessed on 3 August 2024).

In pathology, various techniques identify potential biomarkers that are essential for diagnosis, prognosis, and treatment response. These biomarkers play a pivotal role in clinical trials for disease prediction and treatment monitoring [120]. Histological and molecular biomarkers of RASopathies have been utilized (Figure 3).

These include cellular and tissue structure changes, such as alterations in cell shape and size or extracellular matrix modifications including fibrosis [40,121]. Tissue abnormalities, such as hypertrophic cardiomyopathy, skeletal muscle myopathy, and cutaneous papillomas, are also significant [121,122,123]. Molecular markers involve the presence or absence of specific proteins detectable through immunohistochemistry [124], including Ki-67, p53, AXL, ERK phosphorylation, growth factors and hormone receptors, VEGF, Interleukin-6, CXCR4/CXCL12, calbindin D, S100, p16, and CDKN2A, among others, providing insights into tissue structure, function, and pathological states (Figure 3).

In NF1, specific biomarkers identify clinical and histological features such as neurofibromas [40], café-au-lait spots [125], optic gliomas [126], and Lisch nodules [127], highlighting distinct cellular compositions and tissue characteristics associated with the disease (Figure 3). Additionally, specific biomarkers such as hypercellularity and nuclear atypia in MPNSTs [128]; growth factors, hormone receptors, and signaling pathways in cNFs [120]; and insulin-like growth factor-1 and growth hormone receptors in neoplastic Schwann cells [120], along with ERK phosphorylation; mTOR pathway targets [129]; and histological markers such as calbindin D, S100, and nestin, are crucial for diagnosis, prognosis, patient selection, and treatment assessment [130]. Additional markers such as TLE1, HMGA2, p53, p16, CDKN2A, and miR-204 aid in differentiating tumor types and predicting patient outcomes [131,132,133] (Figure 3). LS shares clinical features with NF1, such as café-au-lait macules and freckling, but lacks neurofibromas [134], and while histological similarities include increased melanin levels [135], specific histological biomarkers unique to LS have not been identified.

Research on biomarkers in NS, NSML, and CS has provided valuable insights for their diagnosis and management. In NS, salivary inflammatory biomarkers such as defensin α1 and thymosin β4 are elevated [136], and males may show specific biomarkers indicating primary testicular insufficiency in Sertoli cells [137]. Histological findings in NS include cellular hyperplasia in skin and connective tissues, with cardiac manifestations suggesting potential histological changes in cardiac tissue [122,138,139]. NSML is characterized by cardiac features such as pulmonary valve stenosis and hypertrophic cardiomyopathy, along with lentigines on the skin [121]. CS presents with dysmorphic facial features, dermatological manifestations, and cardiac anomalies, with histological biomarkers such as abnormal collagen and elastic fibers contributing to its diagnosis and management [1]. Additionally, CS is associated with oncogenic predisposition, featuring both benign and malignant neoplasms such as cutaneous papillomas and rhabdomyosarcoma [123]. Histologically, specific biomarkers for CFC syndrome are not well defined. Clinically, CFC is characterized by cardiac anomalies such as pulmonic stenosis, septal defects, and hypertrophic cardiomyopathy; distinct facial features; and various skin abnormalities include dryness, hyperkeratosis, ichthyosis, keratosis pilaris, and hemangiomas [140]. Thus, histological biomarkers are essential and more reliable for clinical practice in the accurate identification and management of these diseases.

## 5. Physiologic Biomarkers

**Figure 4:**
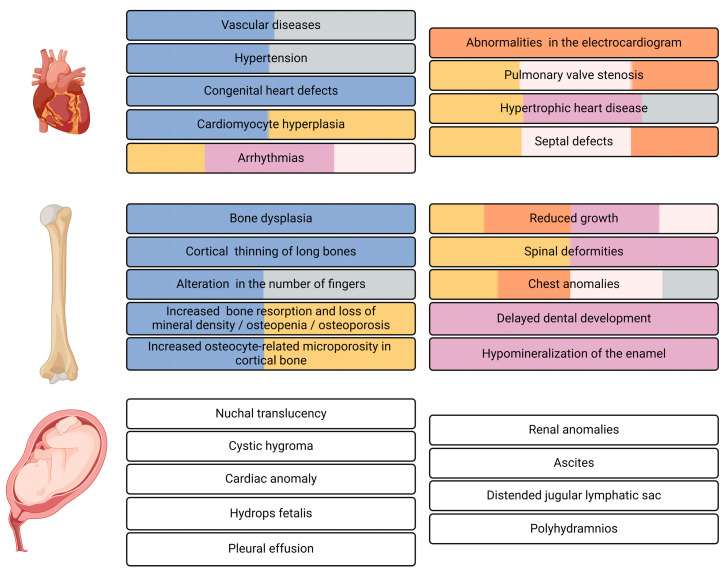
Cardiac, bone, and embryonic RASopathy physiological biomarkers. Each color represents a specific RASopathy. NF1: blue; LS: gray; NS: brown; CS: violet; NSML: orange; CFC: pink. Biomarkers common to all RASopathies are indicated in white. Created with BioRender.com (accessed on 3 August 2024).

In delineating the physiologic markers of RASopathies, distinctions can be made among cardiac, bone, and embryonic indicators (Figure 4).

Biomarkers pertaining to bone physiology, mainly increased bone resorption and loss of mineral density, alterations in the skeleton, reduced growth, alterations in the pectus, and alterations in the number of fingers, have been used in NF1, NS, NSML, CS, CFCS, and LS [22,121,141,142,143,144]. Regarding detection, most bone physiological markers present in RASopathies can be detected through X-rays. On the other hand, the loss of mineral density in bones is detectable by dual-energy X-ray absorptiometry (DXA) or by detecting urine pyridinium levels, since an increase leads to a decrease in mineral density [143].

Biomarkers of cardiac physiology, mainly arrhythmias and electrocardiogram abnormalities, pulmonary valve stenosis, septal defects, and hypertrophic cardiac diseases, are used in the same RASopathies as bone markers [22,38,121,141,143,144]. The detection of these biomarkers can be carried out during gestation through gestational ultrasonography. In adulthood, it is possible to use both electrocardiograms, which detect defects in the heart rhythm, and cardiac magnetic resonance, which can generate images to determine ventricular volume and analyze function as well as ejection fraction and myocardial mass, providing non-invasive mechanisms that make it possible to identify alterations in cardiac physiology [38,142,145]. During pregnancy, suspected prenatal RASopathies can be detected by ultrasound, which allows for the detection of increased nuchal translucency (>95th percentile), cystic hygroma, cardiac anomalies, hydrops fetalis, pleural effusion, renal anomalies, ascites, distended jugular lymphatic sac, and polyhydramnios [146].

Neurodevelopmental and endocrine biomarkers are crucial for diagnosis and management of RASopathies. Common symptoms include developmental delays, cognitive deficits, and structural brain malformations [147]. Neurodevelopmental biomarkers include cognitive and developmental assessments; neuroimaging findings; and variations in brain structure, such as changes in cortical surface area and thickness, as well as subcortical volume effects specific to each syndrome [148]. Conditions such as NF1, NS, and CS are associated with delays in speech and motor skills, learning disabilities, intellectual disabilities, and behavioral issues such as ADHD and autism spectrum disorders [1,147]. Endocrine complications frequently observed in RASopathies include short stature, reduced bone mineral density, and thyroid autoimmunity, with higher anti-TPO antibody levels noted in NS and CFC syndrome [149] (Figure 4). NF1 also presents endocrine challenges such as central precocious puberty, growth hormone deficiency, and hypersecretion, often associated with OPG affecting the hypothalamic–pituitary region [150].

## 6. Conclusions

Future research on biomarkers for RASopathies should focus on expanding metabolomic profiles, conducting longitudinal studies, and employing multi-omic approaches. Key areas include standardizing biomarker measurements, identifying biomarkers across diverse populations, and utilizing advanced imaging techniques. Additionally, exploring epigenetic biomarkers, validating therapeutic ones, and implementing artificial intelligence to analyze complex data and predict disease progression are essential. These efforts will enhance the understanding of disease progression, enable personalized treatment, and support effective clinical implementation, addressing current gaps and improving the management of RASopathies. Additionally, the transition from biomarker discovery to clinical application is a complex process that involves several critical steps and challenges. These include rigorous validation to ensure the biomarker’s reliability and accuracy, as well as extensive clinical trials to establish its predictive value and utility in real-world settings. Furthermore, integrating insights from disciplines such as bioinformatics and systems biology can provide a more comprehensive understanding of the biomarker landscape. These fields contribute valuable tools and methods for analyzing large datasets, identifying patterns, and understanding the molecular mechanisms underlying disease processes. This multidisciplinary approach is essential for overcoming the challenges of biomarker implementation in routine clinical practice, ultimately enhancing patient diagnosis, monitoring, and treatment.

Biomarkers can provide a more objective way to diagnose RASopathies, which can lead to earlier and more effective treatments. They can also help researchers better understand how RASopathies progress, thereby aiding in the development of targeted therapies that are more effective and have fewer side effects. In the study of RASopathies, genetic markers remain the most crucial and widely used biomarkers due to their direct correlation with disease-causing mutations. Non-coding RNAs, such as miRNAs and lncRNAs, are emerging as important biomarkers but are still primarily in the research phase. Protein expression levels are directly linked to the presence and severity of RASopathies but are less frequently used in clinical practice. Phosphorylated proteins, including pAKT, pS6RP, and pERK, show significant promise for monitoring disease progression and treatment efficacy, although further clinical validation is needed. Metabolic markers, while less commonly used, hold potential for monitoring disease severity and complications. Histological and molecular biomarkers also play a vital role in the diagnosis and clinical management of RASopathies, offering critical insights into tissue structure and pathological states. Specific histopathological features, such as the presence of neurofibromas in NF1 or characteristic cardiac malformations in NS and NMSL, as well as dermatological features in CS, provide critical diagnostic clues. The reliability of these biomarkers supports accurate diagnosis, prognosis, and patient selection for targeted treatments. IHC, utilizing antibodies to detect biomarkers such as Ki-67, p53, and S100, plays a crucial role on this process, enabling precise clinical decision making and personalized therapeutic approaches. Physiological biomarkers, including cardiac function assessments and neurodevelopmental evaluations, are important for ongoing monitoring of patients with RASopathies. These non-invasive assessments can help track disease progression and response to treatment, offering valuable information for clinical management. One of the main concerns of current medicine is to offer a personalized or precision approach, which consists of adapting medical treatment according to the patient’s genomic variations, biochemical profile, environment, and lifestyle. There is a significant research gap in translating these biomarkers from research to clinical practice. Limited clinical validation, the need for standardized protocols, and a lack of longitudinal studies are major challenges that need to be addressed. In conclusion, while significant progress has been made in identifying and utilizing biomarkers for RASopathies, much work remains to fully translate these findings into clinical practice. Continued efforts in basic and clinical research will pave the way for more effective and personalized approaches to managing these complex disorders, ultimately improving the health and life expectancy of patients.

## Data Availability

No new data were created in this study. Data sharing is not applicable to this article as no datasets were generated.

## Supplementary Materials

The following supporting information can be downloaded at: https://www.mdpi.com/article/10.3390/ijms25168563/s1.

## References

[1] Rauen K.A. (2013). The RASopathies. Annu. Rev. Genom. Hum. Genet..

[2] Tidyman W.E., Rauen K.A. (2009). The RASopathies: Developmental Syndromes of Ras/MAPK Pathway Dysregulation. Curr. Opin. Genet. Dev..

[3] Rauen K.A. (2022). Defining RASopathy. Dis. Models Mech..

[4] Montero-Bullón J.F., González-Velasco Ó., Isidoro-García M., Lacal J. (2021). Integrated in Silico MS-Based Phosphoproteomics and Network Enrichment Analysis of RASopathy Proteins. Orphanet J. Rare Dis..

[5] Saint-Laurent C., Mazeyrie L., Yart A., Edouard T. (2023). Novel Therapeutic Perspectives in Noonan Syndrome and RASopathies. Eur. J. Pediatr..

[6] Kauffman H., Ahrens-Nicklas R.C., Calderon-Anyosa R.J.C., Ritter A.L., Lin K.Y., Rossano J.W., Quartermain M.D., Banerjee A. (2021). Genotype–Phenotype Association by Echocardiography Offers Incremental Value in Patients with Noonan Syndrome with Multiple Lentigines. Pediatr. Res..

[7] Huckstadt V., Chinton J., Gomez A., Obregon M.G., Gravina L.P. (2021). Noonan Syndrome with Loose Anagen Hair with Variants in the PPP1CB Gene: First Familial Case Reported. Am. J. Med. Genet. Part A.

[8] Wang Q., Cheng S., Fu Y., Yuan H. (2022). Case Report: A de Novo RASopathy-Causing SHOC2 Variant in a Chinese Girl with Noonan Syndrome-like with Loose Anagen Hair. Front. Genet..

[9] Bertola D.R., Castro M.A.A., Yamamoto G.L., Honjo R.S., Ceroni J.R., Buscarilli M.M., Freitas A.B., Malaquias A.C., Pereira A.C., Jorge A.A.L. (2020). Phenotype–Genotype Analysis of 242 Individuals with RASopathies: 18-Year Experience of a Tertiary Center in Brazil. Am. J. Med. Genet. Part C Semin. Med. Genet..

[10] Pabst L., Carroll J., Lo W., Truxal K.V. (2021). Moyamoya Syndrome in a Child with Legius Syndrome: Introducing a Cerebral Vasculopathy to the SPRED1 Phenotype?. Am. J. Med. Genet. Part A.

[11] Scorrano G., David E., Calì E., Chimenz R., La Bella S., Di Ludovico A., Di Rosa G., Gitto E., Mankad K., Nardello R. (2023). The Cardiofaciocutaneous Syndrome: From Genetics to Prognostic–Therapeutic Implications. Genes.

[12] Leoni C., Paradiso F.V., Foschi N., Tedesco M., Pierconti F., Silvaroli S., Diego M.D., Birritella L., Pantaleoni F., Rendeli C. (2022). Prevalence of Bladder Cancer in Costello Syndrome: New Insights to Drive Clinical Decision-Making. Clin. Genet..

[13] Nicholson C.L., Flanagan S., Murati M., Boull C., McGough E., Ameduri R., Weigel B., Maguiness S. (2022). Successful Management of an Arteriovenous Malformation with Trametinib in a Patient with Capillary-Malformation Arteriovenous Malformation Syndrome and Cardiac Compromise. Pediatr. Dermatol..

[14] Báez-Flores J., Rodríguez-Martín M., Lacal J. (2023). The Therapeutic Potential of Neurofibromin Signaling Pathways and Binding Partners. Commun. Biol..

[15] Chen Z., Mo J., Brosseau J.P., Shipman T., Wang Y., Liao C.P., Cooper J.M., Allaway R.J., Gosline S.J.C., Guinney J. (2019). Spatiotemporal Loss of NF1 in Schwann Cell Lineage Leads to Different Types of Cutaneous Neurofibroma Susceptible to Modification by the Hippo Pathway. Cancer Discov..

[16] Lodi M., Boccuto L., Carai A., Cacchione A., Miele E., Colafati G.S., Camassei F.D., de Palma L., de Benedictis A., Ferretti E. (2020). Low-Grade Gliomas in Patients with Noonan Syndrome: Case-Based Review of the Literature. Diagnostics.

[17] Luscan A., Shackleford G.G., Masliah-Planchon J., Laurendeau I., Ortonne N., Varin J., Lallemand F., Leroy K., Dumaine V., Hivelin M. (2014). The Activation of the WNT Signaling Pathway Is a Hallmark in Neurofibromatosis Type 1 Tumorigenesis. Clin. Cancer Res..

[18] Ou F.S., Michiels S., Shyr Y., Adjei A.A., Oberg A.L. (2021). Biomarker Discovery and Validation: Statistical Considerations. J. Thorac. Oncol..

[19] Califf R.M. (2018). Biomarker Definitions and Their Applications. Exp. Biol. Med..

[20] McCann M.R., De la Rosa M.V.G., Rosania G.R., Stringer K.A. (2021). L-Carnitine and Acylcarnitines: Mitochondrial Biomarkers for Precision Medicine. Metabolites.

[21] Gurusamy N., Rajasingh S., Sigamani V., Rajasingh R., Isai D.G., Czirok A., Bittel D., Rajasingh J. (2021). Noonan Syndrome Patient-Specific Induced Cardiomyocyte Model Carrying SOS1 Gene Variant c.1654A>G. Exp. Cell Res..

[22] Jafry M., Sidbury R. (2020). RASopathies. Clin. Dermatol..

[23] Motta M., Pannone L., Pantaleoni F., Bocchinfuso G., Radio F.C., Cecchetti S., Ciolfi A., Di Rocco M., Elting M.W., Brilstra E.H. (2020). Enhanced MAPK1 Function Causes a Neurodevelopmental Disorder within the RASopathy Clinical Spectrum. Am. J. Hum. Genet..

[24] Tamburrino F., Mazzanti L., Scarano E., Gibertoni D., Sirolli M., Zioutas M., Schiavariello C., Perri A., Mantovani A., Rossi C. (2023). Lipid Profile in Noonan Syndrome and Related Disorders: Trend by Age, Sex and Genotype. Front. Endocrinol..

[25] Zenker M. (2022). Clinical Overview on RASopathies. Am. J. Med. Genet. Part C Semin. Med. Genet..

[26] Linglart L., Gelb B.D. (2020). Congenital Heart Defects in Noonan Syndrome: Diagnosis, Management, and Treatment. Am. J. Med. Genet. Part C Semin. Med. Genet..

[27] Tang S., Yuan K., Chen L. (2022). Molecular Biomarkers, Network Biomarkers, and Dynamic Network Biomarkers for Diagnosis and Prediction of Rare Diseases. Fundam. Res..

[28] Legius E., Messiaen L., Wolkenstein P., Pancza P., Avery R.A., Berman Y., Blakeley J., Babovic-Vuksanovic D., Cunha K.S., Ferner R. (2021). Revised Diagnostic Criteria for Neurofibromatosis Type 1 and Legius Syndrome: An International Consensus Recommendation. Genet. Med..

[29] Richards S., Aziz N., Bale S., Bick D., Das S., Gastier-Foster J., Grody W.W., Hegde M., Lyon E., Spector E. (2015). Standards and Guidelines for the Interpretation of Sequence Variants: A Joint Consensus Recommendation of the American College of Medical Genetics and Genomics and the Association for Molecular Pathology. Genet. Med..

[30] Moog U., Felbor U., Has C., Zirn B. (2020). Disorders Caused by Genetic Mosaicism. Dtsch. Arztebl. Int..

[31] Chang C.A., Perrier R., Kurek K.C., Estrada-Veras J., Lehman A., Yip S., Hendson G., Diamond C., Pinchot J.W., Tran J.M. (2021). Novel Findings and Expansion of Phenotype in a Mosaic RASopathy Caused by Somatic KRAS Variants. Am. J. Med. Genet. Part A.

[32] Amani V., Riemondy K.A., Fu R., Griesinger A.M., Grimaldo E., De Sousa G.R., Gilani A., Hemenway M., Foreman N.K., Donson A.M. (2023). Integration of Single-Nuclei RNA-Sequencing, Spatial Transcriptomics and Histochemistry Defines the Complex Microenvironment of NF1-Associated Plexiform Neurofibromas. Acta Neuropathol. Commun..

[33] Douben H., Hoogeveen-Westerveld M., Nellist M., Louwen J., Haan M.K.D., Punt M., Van Ommeren B., Van Unen L., Elfferich P., Kasteleijn E. (2023). Functional Assays Combined with Pre-mRNA-Splicing Analysis Improve Variant Classification and Diagnostics for Individuals with Neurofibromatosis Type 1 and Legius Syndrome. Hum. Mutat..

[34] Koster R., Brandão R.D., Tserpelis D., van Roozendaal C.E.P., van Oosterhoud C.N., Claes K.B.M., Paulussen A.D.C., Sinnema M., Vreeburg M., van der Schoot V. (2021). Pathogenic Neurofibromatosis Type 1 (NF1) RNA Splicing Resolved by Targeted RNAseq. npj Genom. Med..

[35] Hartung A.M., Swensen J., Uriz I.E., Lapin M., Kristjansdottir K., Petersen U.S.S., Bang J.M.V., Guerra B., Andersen H.S., Dobrowolski S.F. (2016). The Splicing Efficiency of Activating HRAS Mutations Can Determine Costello Syndrome Phenotype and Frequency in Cancer. PLoS Genet..

[36] Devonshire A.S., Sanders R., Wilkes T.M., Taylor M.S., Foy C.A., Huggett J.F. (2013). Application of next Generation qPCR and Sequencing Platforms to mRNA Biomarker Analysis. Methods.

[37] Hanses U., Kleinsorge M., Roos L., Yigit G., Li Y., Barbarics B., El-Battrawy I., Lan H., Tiburcy M., Hindmarsh R. (2020). Intronic CRISPR Repair in a Preclinical Model of Noonan Syndrome-Associated Cardiomyopathy. Circulation.

[38] Drenckhahn J.D., Nicin L., Akhouaji S., Krück S., Blank A.E., Schänzer A., Yörüker U., Jux C., Tombor L., Abplanalp W. (2023). Cardiomyocyte Hyperplasia and Immaturity but Not Hypertrophy Are Characteristic Features of Patients with RASopathies. J. Mol. Cell. Cardiol..

[39] Coccia E., Valeri L., Zuntini R., Caraffi S.G., Peluso F., Pagliai L., Vezzani A., Pietrangiolillo Z., Leo F., Melli N. (2023). Prenatal Clinical Findings in RASA1-Related Capillary Malformation-Arteriovenous Malformation Syndrome. Genes.

[40] Brosseau J.P., Sathe A.A., Wang Y., Nguyen T., Glass D.A., Xing C., Le L.Q. (2021). Human Cutaneous Neurofibroma Matrisome Revealed by Single-Cell RNA Sequencing. Acta Neuropathol. Commun..

[41] Imada E.L., Strianese D., Edward D.P., alThaqib R., Price A., Arnold A., Al-Hussain H., Marchionni L., Rodriguez F.J. (2022). RNA-Sequencing Highlights Differential Regulated Pathways Involved in Cell Cycle and Inflammation in Orbitofacial Neurofibromas. Brain Pathol..

[42] Yan H., Bu P. (2021). Non-Coding RNA in Cancer. Essays Biochem..

[43] de Carvalho J.B., de Morais G.L., Vieira T.C.d.S., Rabelo N.C., Llerena J.C., de Carvalho Gonzalez S.M., de Vasconcelos A.T.R. (2019). miRNA Genetic Variants Alter Their Secondary Structure and Expression in Patients with RASopathies Syndromes. Front. Genet..

[44] Yu T.T., Xu Q.F., Li S.Y., Huang H.J., Dugan S., Shao L., Roggenbuck J.A., Liu X.T., Liu H.Z., Hirsch B.A. (2021). Deletion at an 1q24 Locus Reveals a Critical Role of Long Noncoding RNA DNM3OS in Skeletal Development. Cell Biosci..

[45] Amirnasr A., Verdijk R.M., van Kuijk P.F., Kartal P., Vriends A.L.M., French P.J., van Royen M.E., Taal W., Sleijfer S., Wiemer E.A.C. (2020). Deregulated microRNAs in Neurofibromatosis Type 1 Derived Malignant Peripheral Nerve Sheath Tumors. Sci. Rep..

[46] Khosravi T., Oladnabi M. (2023). The Role of miRNAs and lncRNAs in Neurofibromatosis Type 1. J. Cell. Biochem..

[47] Nix J.S., Yuan M., Imada E.L., Ames H., Marchionni L., Gutmann D.H., Rodriguez F.J. (2021). Global microRNA Profiling Identified miR-10b-5p as a Regulator of Neurofibromatosis 1 (NF1)-Glioma Migration. Neuropathol. Appl. Neurobiol..

[48] Mulero-Navarro S., Sevilla A., Roman A.C., Lee D.F., D’Souza S.L., Pardo S., Riess I., Su J., Cohen N., Schaniel C. (2015). Myeloid Dysregulation in a Human Induced Pluripotent Stem Cell Model of PTPN11-Associated Juvenile Myelomonocytic Leukemia. Cell Rep..

[49] Herman A.B., Tsitsipatis D., Gorospe M. (2022). Integrated lncRNA Function upon Genomic and Epigenomic Regulation. Mol. Cell.

[50] Tritto V., Ferrari L., Esposito S., Zuccotti P., Bianchessi D., Natacci F., Saletti V., Eoli M., Riva P. (2019). Non-Coding RNA and Tumor Development in Neurofibromatosis Type 1: ANRIL Rs2151280 Is Associated with Optic Glioma Development and a Mild Phenotype in Neurofibromatosis Type 1 Patients. Genes.

[51] Gyorffy B., Schafer R. (2010). Biomarkers Downstream of RAS: A Search for Robust Transcriptional Targets. Curr. Cancer Drug Targets.

[52] Campbell S.L., Philips M.R. (2021). Post-Translational Modification of RAS Proteins. Curr. Opin. Struct. Biol..

[53] Jang H.H. (2018). Regulation of Protein Degradation by Proteasomes in Cancer. J. Cancer Prev..

[54] Dohlman H.G., Campbell S.L. (2019). Regulation of Large and Small G Proteins by Ubiquitination. J. Biol. Chem..

[55] Kim S.E., Yoon J.Y., Jeong W.J., Jeon S.H., Park Y., Yoon J.B., Park Y.N., Kim H., Choi K.Y. (2009). H-Ras Is Degraded by Wnt/β-Catenin Signaling via β-TrCP-Mediated Polyubiquitylation. J. Cell Sci..

[56] Yoshino H., Yin G., Kawaguchi R., Popov K.I., Temple B., Sasaki M., Kofuji S., Wolfe K., Kofuji K., Okumura K. (2019). Identification of Lysine Methylation in the Core GTPase Domain by GoMADScan. PLoS ONE.

[57] Castellano E., Santos E. (2011). Functional Specificity of Ras Isoforms: So Similar but so Different. Genes Cancer.

[58] Ratner N., Miller S.J. (2015). A RASopathy Gene Commonly Mutated in Cancer: The Neurofibromatosis Type 1 Tumour Suppressor. Nat. Rev. Cancer.

[59] Dasgupta B., Yi Y., Chen D.Y., Weber J.D., Gutmann D.H. (2005). Proteomic Analysis Reveals Hyperactivation of the Mammalian Target of Rapamycin Pathway in Neurofibromatosis 1-Associated Human and Mouse Brain Tumors. Cancer Res..

[60] Bergoug M., Doudeau M., Godin F., Mosrin C., Vallée B., Bénédetti H. (2020). Neurofibromin Structure, Functions and Regulation. Cells.

[61] McFall T., Stites E.C. (2021). Identification of RAS Mutant Biomarkers for EGFR Inhibitor Sensitivity Using a Systems Biochemical Approach. Cell Rep..

[62] Koliou X., Fedonidis C., Kalpachidou T., Mangoura D. (2016). Nuclear Import Mechanism of Neurofibromin for Localization on the Spindle and Function in Chromosome Congression. J. Neurochem..

[63] Feng L., Yunoue S., Tokuo H., Ozawa T., Zhang D., Patrakitkomjorn S., Ichimura T., Saya H., Araki N. (2004). PKA Phosphorylation and 14-3-3 Interaction Regulate the Function of Neurofibromatosis Type I Tumor Suppressor, Neurofibromin. FEBS Lett..

[64] Harder A., Rosche M., Reuß D.E., Holtkamp N., Uhlmann K., Friedrich R., Mautner V.F., Von Deimling A. (2004). Methylation Analysis of the Neurofibromatosis Type 1 (NF1) Promoter in Peripheral Nerve Sheath Tumours. Eur. J. Cancer.

[65] Beauclair G., Bridier-Nahmias A., Zagury J.F., Säb A., Zamborlini A. (2015). JASSA: A Comprehensive Tool for Prediction of SUMOylation Sites and SIMs. Bioinformatics.

[66] Yan W., Markegard E., Dharmaiah S., Urisman A., Drew M., Esposito D., Scheffzek K., Nissley D.V., McCormick F., Simanshu D.K. (2020). Structural Insights into the SPRED1-Neurofibromin-KRAS Complex and Disruption of SPRED1-Neurofibromin Interaction by Oncogenic EGFR. Cell Rep..

[67] Pandit B., Sarkozy A., Pennacchio L.A., Carta C., Oishi K., Martinelli S., Pogna E.A., Schackwitz W., Ustaszewska A., Landstrom A. (2007). Gain-of-Function RAF1 Mutations Cause Noonan and LEOPARD Syndromes with Hypertrophic Cardiomyopathy. Nat. Genet..

[68] Razzaque M.A., Nishizawa T., Komoike Y., Yagi H., Furutani M., Amo R., Kamisago M., Momma K., Katayama H., Nakagawa M. (2007). Germline Gain-of-Function Mutations in RAF1 Cause Noonan Syndrome. Nat. Genet..

[69] Heidorn S.J., Milagre C., Whittaker S., Nourry A., Niculescu-Duvas I., Dhomen N., Hussain J., Reis-Filho J.S., Springer C.J., Pritchard C. (2010). Kinase-Dead BRAF and Oncogenic RAS Cooperate to Drive Tumor Progression through CRAF. Cell.

[70] Roberts A.E., Allanson J.E., Tartaglia M., Gelb B.D. (2013). Noonan Syndrome. Lancet.

[71] Galperin E., Wilson P., Abdelmoti L., Norcross R., Palayam M. (2022). Proteins of the Ubiquitin System in the Shoc2—ERK1/2 Signaling Axis and Noonan-like Syndrome with Loose Anagen Hair (NSLAH) RASopathy. FASEB J..

[72] Bivona T.G., Quatela S.E., Bodemann B.O., Ahearn I.M., Soskis M.J., Mor A., Miura J., Wiener H.H., Wright L., Saba S.G. (2006). PKC Regulates a Farnesyl-Electrostatic Switch on K-Ras That Promotes Its Association with Bcl-XL on Mitochondria and Induces Apoptosis. Mol. Cell.

[73] Choi B.H., Philips M.R., Chen Y., Lu L., Dai W. (2018). K-Ras Lys-42 Is Crucial for Its Signaling, Cell Migration, and Invasion. J. Biol. Chem..

[74] Ottaiano A., Normanno N., Facchini S., Cassata A., Nappi A., Romano C., Silvestro L., Stefano A.D., Rachiglio A.M., Roma C. (2020). Study of Ras Mutations’ Prognostic Value in Metastatic Colorectal Cancer: Storia Analysis. Cancers.

[75] Simão S., Agostinho R.R., Martínez-Ruiz A., Araújo I.M. (2023). Regulation of Ras Signaling by S-Nitrosylation. Antioxidants.

[76] Martin-Vega A., Cobb M.H. (2023). Navigating the ERK1/2 MAPK Cascade. Biomolecules.

[77] Yang X., You J., Luo W., Yue J., Ma L., Xiao W., Zhu D., Wu Z., Wang D., Nadiminty N. (2010). The N-Terminal Kinase Suppressor of Ras Complex Has a Weak Nucleoside Diphosphate Kinase Activity. Thorac. Cancer.

[78] Hahn A., Lauriol J., Thul J., Behnke-Hall K., Logeswaran T., Schänzer A., Böğürcü N., Garvalov B.K., Zenker M., Gelb B.D. (2015). Rapidly Progressive Hypertrophic Cardiomyopathy in an Infant with Noonan Syndrome with Multiple Lentigines: Palliative Treatment with a Rapamycin Analog. Am. J. Med. Genet. Part A.

[79] Ranza E., Guimier A., Verloes A., Capri Y., Marques C., Auclair M., Mathieu-Dramard M., Morin G., Thevenon J., Faivre L. (2020). Overlapping Phenotypes between SHORT and Noonan Syndromes in Patients with PTPN11 Pathogenic Variants. Clin. Genet..

[80] Endo M., Yamamoto H., Setsu N., Kohashi K., Takahashi Y., Ishii T., Iida K.I., Matsumoto Y., Hakozaki M., Aoki M. (2013). Prognostic Significance of AKT/mTOR and MAPK Pathways and Antitumor Effect of mTOR Inhibitor in NF1-Related and Sporadic Malignant Peripheral Nerve Sheath Tumors. Clin. Cancer Res..

[81] Solares I., Viñal D., Morales-Conejo M., Rodriguez-Salas N., Feliu J. (2021). Novel Molecular Targeted Therapies for Patients with Neurofibromatosis Type 1 with Inoperable Plexiform Neurofibromas: A Comprehensive Review. ESMO Open.

[82] Ugwu N., Cheraghlou S., Ko C.J., Cohen J.M. (2023). Incidence, Survival, and Prognostic Factors Associated with Malignant Nodular Hidradenoma in the United States. J. Am. Acad. Dermatol..

[83] Ullrich N.J., Prabhu S.P., Reddy A.T., Fisher M.J., Packer R., Goldman S., Robison N.J., Gutmann D.H., Viskochil D.H., Allen J.C. (2020). A Phase II Study of Continuous Oral mTOR Inhibitor Everolimus for Recurrent, Radiographic-Progressive Neurofibromatosis Type 1-Associated Pediatric Low-Grade Glioma: A Neurofibromatosis Clinical Trials Consortium Study. Neuro Oncol..

[84] Miyoshi K., Wakioka T., Nishinakamura H., Kamio M., Yang L., Inoue M., Hasegawa M., Yonemitsu Y., Komiya S., Yoshimura A. (2004). The Sprouty-Related Protein, Spred, Inhibits Cell Motility, Metastasis, and Rho-Mediated Actin Reorganization. Oncogene.

[85] Brown J.A., Diggs-Andrews K.A., Gianino S.M., Gutmann D.H. (2012). Neurofibromatosis-1 Heterozygosity Impairs CNS Neuronal Morphology in a cAMP/PKA/ROCK-Dependent Manner. Mol. Cell. Neurosci..

[86] Ozawa T., Araki N., Yunoue S., Tokuo H., Feng L., Patrakitkomjorn S., Hara T., Ichikawa Y., Matsumoto K., Fuji K. (2005). The Neurofibromatosis Type 1 Gene Product Neurofibromin Enhances Cell Motility by Regulating Actin Filament Dynamics via the Rho-ROCK-LIMK2-Cofilin Pathway. J. Biol. Chem..

[87] Langdon Y., Tandon P., Paden E., Duddy J., Taylor J.M., Conlon F.L. (2012). SHP-2 Acts via ROCK to Regulate the Cardiac Actin Cytoskeleton. Development.

[88] Chen M., Lu L., Cheng D., Zhang J., Liu X., Zhang J., Zhang T. (2023). Icariin Promotes Osteogenic Differentiation in a Cell Model with NF1 Gene Knockout by Activating the cAMP/PKA/CREB Pathway. Molecules.

[89] Mazuelas H., Magallón-Lorenz M., Uriarte-Arrazola I., Negro A., Rosas I., Blanco I., Castellanos E., Lázaro C., Gel B., Carrió M. (2024). Unbalancing cAMP and Ras/MAPK Pathways as a Therapeutic Strategy for Cutaneous Neurofibromas. JCI Insight.

[90] Biayna J., Mazuelas H., Gel B., Terribas E., Dumbovic G., Rosas I., Fernández-Rodriguez J., Blanco I., Castellanos E., Carrió M. (2021). Using Antisense Oligonucleotides for the Physiological Modulation of the Alternative Splicing of NF1 Exon 23a during PC12 Neuronal Differentiation. Sci. Rep..

[91] Machado Almeida P., Lago Solis B., Stickley L., Feidler A., Nagoshi E. (2021). Neurofibromin 1 in Mushroom Body Neurons Mediates Circadian Wake Drive through Activating cAMP–PKA Signaling. Nat. Commun..

[92] Xue C., Yao Q., Gu X., Shi Q., Yuan X., Chu Q., Bao Z., Lu J., Li L. (2023). Evolving Cognition of the JAK-STAT Signaling Pathway: Autoimmune Disorders and Cancer. Signal Transduct. Target. Ther..

[93] Miot H.A., Criado P.R., de Castro C.C.S., Ianhez M., Talhari C., Ramos P.M. (2023). JAK-STAT Pathway Inhibitors in Dermatology. An. Bras. Dermatol..

[94] Erdogan F., Radu T.B., Orlova A., Qadree A.K., de Araujo E.D., Israelian J., Valent P., Mustjoki S.M., Herling M., Moriggl R. (2022). JAK-STAT Core Cancer Pathway: An Integrative Cancer Interactome Analysis. J. Cell. Mol. Med..

[95] Chan G., Kalaitzidis D., Neel B.G. (2008). The Tyrosine Phosphatase Shp2 (PTPN11) in Cancer. Cancer Metastasis Rev..

[96] Seif F., Khoshmirsafa M., Aazami H., Mohsenzadegan M., Sedighi G., Bahar M. (2017). The Role of JAK-STAT Signaling Pathway and Its Regulators in the Fate of T Helper Cells. Cell Commun. Signal..

[97] Wu J., Keng V.W., Patmore D.M., Kendall J.J., Patel A.V., Jousma E., Jessen W.J., Choi K., Tschida B.R., Silverstein K.A.T. (2016). Insertional Mutagenesis Identifies a STAT3/Arid1b/β-Catenin Pathway Driving Neurofibroma Initiation. Cell Rep..

[98] Ma S., Meng Z., Chen R., Guan K.-L. (2019). The Hippo Pathway: Biology and Pathophysiology. Annu. Rev. Biochem..

[99] O’Neill E. (2017). Ras and the Hippo Pathway in Cancer. Conquering RAS: From Biology to Cancer Therapy.

[100] Faden D.L., Asthana S., Tihan T., De Risi J., Kliot M. (2017). Whole Exome Sequencing of Growing and Non-Growing Cutaneous Neurofibromas from a Single Patient with Neurofibromatosis Type 1. PLoS ONE.

[101] Vélez-Reyes G.L., Koes N., Ryu J.H., Kaufmann G., Berner M., Weg M.T., Wolf N.K., Rathe S.K., Ratner N., Moriarity B.S. (2021). Transposon Mutagenesis-Guided Crispr/Cas9 Screening Strongly Implicates Dysregulation of Hippo/Yap Signaling in Malignant Peripheral Nerve Sheath Tumor Development. Cancers.

[102] Zhao H., Ming T., Tang S., Ren S., Yang H., Liu M., Tao Q., Xu H. (2022). Wnt Signaling in Colorectal Cancer: Pathogenic Role and Therapeutic Target. Mol. Cancer.

[103] Jeong W.J., Ro E.J., Choi K.Y. (2018). Interaction between Wnt/β-Catenin and RAS-ERK Pathways and an Anti-Cancer Strategy via Degradations of β-Catenin and RAS by Targeting the Wnt/β-Catenin Pathway. npj Precis. Oncol..

[104] He K., Gan W.J. (2023). Wnt/β-Catenin Signaling Pathway in the Development and Progression of Colorectal Cancer. Cancer Manag. Res..

[105] Tompa M., Nagy A., Komoly S., Kalman B. (2019). Wnt Pathway Markers in Molecular Subgroups of Glioblastoma. Brain Res..

[106] Noda S., Takahashi A., Hayashi T., Tanuma S.I., Hatakeyama M. (2016). Determination of the Catalytic Activity of LEOPARD Syndrome-Associated SHP2 Mutants toward Parafibromin, a Bona Fide SHP2 Substrate Involved in Wnt Signaling. Biochem. Biophys. Res. Commun..

[107] Derynck R., Budi E.H. (2019). Specificity, Versatility, and Control of TGF-b Family Signaling. Sci. Signal..

[108] Tzavlaki K., Moustakas A. (2020). TGF-Β Signaling. Biomolecules.

[109] Torres K.C.L., Lima G., Simões e Silva A.C., Lubambo I., Rodrigues L.O., Rodrigues L., Silveira K.D., Vieira É.L.M., Romano-Silva M.A., Miranda D.M. (2016). Immune Markers in the RASopathy Neurofibromatosis Type 1. J. Neuroimmunol..

[110] Ju Y., Park J.S., Kim D., Kim B., Lee J.H., Nam Y., Yoo H.W., Lee B.H., Han Y.M. (2020). SHP2 Mutations Induce Precocious Gliogenesis of Noonan Syndrome-Derived iPSCs during Neural Development in Vitro. Stem Cell Res. Ther..

[111] Jin Q., Ma R.C.W. (2021). Metabolomics in Diabetes and Diabetic Complications: Insights from Epidemiological Studies. Cells.

[112] Occelli C., Levraut J., Pourcher T. (2024). Metabolomics, the Future of Biomarkers?. Eur. J. Emerg. Med..

[113] Martins A.S., Jansen A.K., Rodrigues L.O.C., Matos C.M., Souza M.L.R., Miranda D.M., de Rezende N.A. (2018). Increased Insulin Sensitivity in Individuals with Neurofibromatosis Type 1. Arch. Endocrinol. Metab..

[114] Martins A.S., Jansen A.K., Rodrigues L.O.C., Matos C.M., Souza M.L.R., de Souza J.F., de Fátima Haueisen Sander Diniz M., Barreto S.M., Diniz L.M., de Rezende N.A. (2016). Lower Fasting Blood Glucose in Neurofibromatosis Type 1. Endocr. Connect..

[115] Tritz R., Benson T., Harris V., Hudson F.Z., Mintz J., Zhang H., Kennard S., Chen W., Stepp D.W., Csanyi G. (2021). Nf1 Heterozygous Mice Recapitulate the Anthropometric and Metabolic Features of Human Neurofibromatosis Type 1. Transl. Res..

[116] Noronha R.M., Villares S.M.F., Torres N., Quedas E.P.S., Homma T.K., Albuquerque E.V.A., Moraes M.B., Funari M.F.A., Bertola D.R., Jorge A.A.L. (2021). Noonan Syndrome Patients beyond the Obvious Phenotype: A Potential Unfavorable Metabolic Profile. Am. J. Med. Genet. Part A.

[117] Fahrner J.A., Frazier A., Bachir S., Walsh M.F., Applegate C.D., Thompson R., Halushka M.K., Murphy A.M., Gunay-Aygun M. (2012). A Rasopathy Phenotype with Severe Congenital Hypertrophic Obstructive Cardiomyopathy Associated with a PTPN11 Mutation and a Novel Variant in SOS1. Am. J. Med. Genet. A.

[118] Gripp K.W., Kawame H., Viskochil D.H., Nicholson L. (2004). Elevated Catecholamine Metabolites in Patients with Costello Syndrome. Am. J. Med. Genet..

[119] Marciano D.P., Snyder M.P., D’Alessandro A. (2019). Personalized Metabolomics. High-Throughput Metabolomics: Methods and Protocols.

[120] Wallis D., Stemmer-Rachamimov A., Adsit S., Korf B., Pichard D., Blakeley J., Sarin K.Y. (2021). Status and Recommendations for Incorporating Biomarkers for Cutaneous Neurofibromas Into Clinical Research. Neurology.

[121] Hilal N., Chen Z., Chen M.H., Choudhury S. (2023). RASopathies and Cardiac Manifestations. Front. Cardiovasc. Med..

[122] Meier A.B., Raj Murthi S., Rawat H., Toepfer C.N., Santamaria G., Schmid M., Mastantuono E., Schwarzmayr T., Berutti R., Cleuziou J. (2022). Cell Cycle Defects Underlie Childhood-Onset Cardiomyopathy Associated with Noonan Syndrome. iScience.

[123] Siegel D.H., Mann J.A., Krol A.L., Rauen K.A. (2012). Dermatological Phenotype in Costello Syndrome: Consequences of Ras Dysregulation in Development. Br. J. Dermatol..

[124] Magaki S., Hojat S.A., Wei B., So A., Yong W.H. (2019). An Introduction to the Performance of Immunohistochemistry. Methods in Molecular Biology.

[125] Peltonen S., Kallionpää R.A., Peltonen J. (2017). Neurofibromatosis Type 1 (NF1) Gene: Beyond Café Au Lait Spots and Dermal Neurofibromas. Exp. Dermatol..

[126] Helfferich J., Nijmeijer R., Brouwer O.F., Boon M., Fock A., Hoving E.W., Meijer L., den Dunnen W.F.A., de Bont E.S.J.M. (2016). Neurofibromatosis Type 1 Associated Low Grade Gliomas: A Comparison with Sporadic Low Grade Gliomas. Crit. Rev. Oncol. Hematol..

[127] Ozarslan B., Russo T., Argenziano G., Santoro C., Piccolo V. (2021). Cutaneous Findings in Neurofibromatosis Type 1. Cancers.

[128] Miettinen M.M., Antonescu C.R., Fletcher C.D.M., Kim A., Lazar A.J., Quezado M.M., Reilly K.M., Stemmer-Rachamimov A., Stewart D.R., Viskochil D. (2017). Histopathologic Evaluation of Atypical Neurofibromatous Tumors and Their Transformation into Malignant Peripheral Nerve Sheath Tumor in Patients with Neurofibromatosis 1—A Consensus Overview. Hum. Pathol..

[129] Kim A., Dombi E., Tepas K., Fox E., Martin S., Wolters P., Balis F.M., Jayaprakash N., Turkbey B., Muradyan N. (2013). Phase I Trial and Pharmacokinetic Study of Sorafenib in Children with Neurofibromatosis Type I and Plexiform Neurofibromas. Pediatr. Blood Cancer.

[130] Meyerholz D.K., Ofori-Amanfo G.K., Leidinger M.R., Goeken J.A., Khanna R., Sieren J.C., Darbro B.W., Quelle D.E., Weimer J.M. (2017). Immunohistochemical Markers for Prospective Studies in Neurofibromatosis-1 Porcine Models. J. Histochem. Cytochem..

[131] Guedes-Corrêa J., Cardoso R. (2018). Immunohistochemical Markers for Schwannomas, Neurofibromas and Malignant Peripheral Nerve Sheath Tumors—What Can the Recent Literature Tell Us?. Arq. Bras. Neurocir. Braz. Neurosurg..

[132] Martin E., Acem I., Grünhagen D.J., Bovée J.V.M.G., Verhoef C. (2020). Prognostic Significance of Immunohistochemical Markers and Genetic Alterations in Malignant Peripheral Nerve Sheath Tumors: A Systematic Review. Front. Oncol..

[133] Johansson G., Peng P.C., Huang P.Y., Chien H.F., Hua K.T., Kuo M.L., Chen C.T., Lee M.J. (2014). Soluble AXL: A Possible Circulating Biomarker for Neurofibromatosis Type 1 Related Tumor Burden. PLoS ONE.

[134] Brems H., Pasmant E., Van Minkelen R., Wimmer K., Upadhyaya M., Legius E., Messiaen L. (2012). Review and Update of SPRED1 Mutations Causing Legius Syndrome. Hum. Mutat..

[135] Zhang J., Li M., Yao Z. (2016). Molecular Screening Strategies for NF1-like Syndromes with Café-Au-Lait Macules (Review). Mol. Med. Rep..

[136] Guglielmi F., Kirschner F., Staderini E., Iavarone F., Fiorino A., Gallenzi P. (2023). Proteomic Analysis of Salivary Inflammatory Biomarkers of Developmental Gingival Enlargements in Patients with West and Noonan Syndromes: A Preliminary Pilot Single-Center Retrospective Study. Eur. Rev. Med. Pharmacol. Sci..

[137] Moniez S., Pienkowski C., Lepage B., Hamdi S., Daudin M., Oliver I., Jouret B., Cartault A., Diene G., Verloes A. (2018). Noonan Syndrome Males Display Sertoli Cell-Specific Primary Testicular Insufficiency. Eur. J. Endocrinol..

[138] Sun L., Xie Y.M., Wang S.S., Zhang Z.W. (2022). Cardiovascular Abnormalities and Gene Mutations in Children with Noonan Syndrome. Front. Genet..

[139] Pierpont E.I., Hudock R.L., Foy A.M., Semrud-Clikeman M., Pierpont M.E., Berry S.A., Shanley R., Rubin N., Sommer K., Moertel C.L. (2018). Social Skills in Children with RASopathies: A Comparison of Noonan Syndrome and Neurofibromatosis Type 1. J. Neurodev. Disord..

[140] Pierpont M.E.M., Magoulas P.L., Adi S., Kavamura M.I., Neri G., Noonan J., Pierpont E.I., Reinker K., Roberts A.E., Shankar S. (2014). Cardio-Facio-Cutaneous Syndrome: Clinical Features, Diagnosis, and Management Guidelines. Pediatrics.

[141] Hebron K.E., Hernandez E.R., Yohe M.E. (2022). The RASopathies: From Pathogenetics to Therapeutics. DMM Dis. Models Mech..

[142] Palit A., Inamadar A.C. (2022). RASopathies: Dermatologists’ Viewpoints. Indian J. Dermatol. Venereol. Leprol..

[143] Fowlkes J.L., Thrailkill K.M., Bunn R.C. (2021). RASopathies: The Musculoskeletal Consequences and Their Etiology and Pathogenesis. Bone.

[144] Lioncino M., Monda E., Verrillo F., Moscarella E., Calcagni G., Drago F., Marino B., Digilio M.C., Putotto C., Calabrò P. (2022). Hypertrophic Cardiomyopathy in RASopathies: Diagnosis, Clinical Characteristics, Prognostic Implications, and Management. Heart Fail. Clin..

[145] Delogu A.B., Blandino R., Leoni C., Tartaglia M., Zampino G. (2023). RASopathies and Sigmoid-Shaped Ventricular Septum Morphology: Evidence of a Previously Unappreciated Cardiac Phenotype. Pediatr. Res..

[146] Mangels R., Blumenfeld Y.J., Homeyer M., Mrazek-Pugh B., Hintz S.R., Hudgins L. (2021). RASopathies: A Significant Cause of Polyhydramnios?. Prenat. Diagn..

[147] Kim Y.E., Baek S.T. (2019). Neurodevelopmental Aspects of RASopathies. Mol. Cells.

[148] McGhee C.A., Honari H., Siqueiros-Sanchez M., Serur Y., van Staalduinen E.K., Stevenson D., Bruno J.L., Raman M.M., Green T. (2024). Influences of RASopathies on Neuroanatomical Variation in Children. Biol. Psychiatry Cogn. Neurosci. Neuroimaging.

[149] Siano M.A., Pivonello R., Salerno M., Falco M., Mauro C., De Brasi D., Klain A., Sestito S., De Luca A., Pinna V. (2022). Endocrine System Involvement in Patients with RASopathies: A Case Series. Front. Endocrinol..

[150] Bizzarri C., Bottaro G. (2015). Endocrine Implications of Neurofibromatosis 1 in Childhood. Horm. Res. Paediatr..

