# Supplementary material for: Biomarker Landscape in RASopathies
Source: Int J Mol Sci. 2024 Aug 6;25(16):8563. doi: 10.3390/ijms25168563 (PMC11354534; doi:10.3390/ijms25168563)
Supplement: Supplementary file 1 [file ijms-25-08563-s001.zip › ijms-3118150-supplementary.pdf]

RASopathy	Aim	Genetic mutation	Effect	Reference
NF1	Assessment of the effects of NF1 variants on pre-mRNA splicing by exon trap constructs	c.6580–2 A > G	Exon skipping and insertion of a 17nt intron sequence	33
		c.1260+ 1604A > G	Inclusion of a cassette exon with 42 bp of intron 11 and introduction of a premature stop codon	
		c.2252 G > T (p.(Gly751Val))	Exon 19 skipping	
		c.556 G > T (p.(Asp186Tyr))	Exon 5 skipping and inclusion of a cryptic intron	
		NF1 c.288+3 A>T, r.205_288del, p.(Arg69_Gly96del)	NF1 pre-mRNA splicing and in-frame deletion	
		c.3829G>C, p.(Gly1277Arg)	Disruption of RAS GAP activity	
	Detection of pathogenic RNA splicing and associated pathogenic DNA variants	c.6580–2 A > G	Exon 43 skipping and insertion of a 17nt intron sequence	34
		c.1260+1604A > G	Introduction of a cassette exon including 42 bp of intron 11 and introduction of a premature stop codon	
		c.2252 G > T (p.(Gly751Val))	Exon 19 skipping and production of a nonsense-mediated decay (NMD)	
		c.556 G > T (p.(Asp186Tyr)	Skipping of exon 5 and the inclusion of a cryptic intron between exons 4 and 5	
		c.5749+332 A > G	Inclusion of a 177 bp cassette exon in intron 38 and insertion of premature stop codon	
CS	Analysis of exon 2 skipping levels in *HRAS* pathogenic variant	c.35_36GC>TG	Exon 2 skipping and disruption function of a critical Exonic Splicing Enhancer and creation of an Exonic Splicing Silencer	35

Supplementary Table S1. mRNA as biomarkers.

**RASopathy**	**Aim**	**Genes**	**Deregulation**	**Reference**
NF1	Study of ECM composition in cNFs compared to normal skin	Collagen VI genes *(COL6A1, COL6A2, COL6A3)*	Overexpression	40
	Pathway enrichment study in orbitofacial NFs	*PRRX1*	Overexpression	41
		CXCL14		
		CCDC80		
		MMP27		
		CCN5 (WISP2)		
		EDNRA		
		XG		
		MME		
		FAT4		
		OSR1		
		AR		
		GAP43	Underexpression	
		MEST		
		SLCO2B1		
		SDC3		
		CIITA		
		SERPINA3		
		SH3TC1		
		ITGB8		
		SLC11A1		
		HOXB7		
		CSF3R		
	Tumor environment of PNFs study by combining scRNA-seq and ST	*LAMA2*	Overexpression in endoneurial fibroblasts	32
		ABCA10		
		AL445250		
		ABCA9		
		EBF2		
		BNC2	Overexpression in perineurial fibroblasts	
		SLC22A3		
		PDZRN4		
		TENM2		
		SORBS		
		SLIT2	Overexpression in epineurial fibroblasts	
		DCLK1		
		PRRX1		
		NAV3		
		RORA		
		TLR2	Overexpression in myeloid subpopulations	
		CD86		
		immune-c13		
		immune c7		
NS	Transcriptomic clustering of WT and *LZTR1*-deficient iPSC-CMs for KEGG pathway enrichment analysis	444 genes	Overexpression	37
		777 genes	Underexpression	
NSML	Differential gene expression analysis of a NSML patient cardiomyocytes	200 genes	Overexpression	38
		481 genes	Underexpression	
CM-AVM	Pathogenic variants effect on gene expression	*RASA1*	Overexpression	39
		RAS/MAPK genes		

Supplementary Table S2. mRNA potential biomarkers.

ncRNA	RASopathy	Aim	Technique	RNA name	Function or Target	Dysregulation	Reference
**miRNA**	RASopathy-phenotype	Detection of miRNA pathogenic variants	WES	hsa-miR-146a-3p, hsa-miR-196a-3p, hsa-miR-548l, hsa-miR-449b-5p, hsa-miR-575, and hsa-miR499a-3p,			45
	NF1	MPNSTs downregulation and upregulation of miRNAs compared to PNFs	RT-qPCR	miR-23/27/24		Down	47
				miR29b1/29a		Down	47
				miR-143-3p	Cancer genes	Down	47
				miR-145-5p	Cancer genes	Down	47
				let7a/b	Cell migration and invasion genes	Down	47
				miR135b	Inducing tumor progression; Wnt negative regulators	Up	47
				miR-889	Wnt negative regulators	Up	47
				miR-493		Up	47
				miR-433		Up	47
				miR-541		Up	47
		MPNSTs downregulation and upregulation of miRNAs compared to PNFs	RT-qPCR	miR-135b	APC	Up	48
				miR-449a	CCND1 and HDAC1	Up	48
				miR-210	HOXA1, HOXA9, HOXA3, and E2F3	Up	48
				miR-301b	PTEN	Up	48
				miR-301a		Up	48
				miR-9	CDH1 and CDX2	Up	48
				miR-130b		Up	48
				miR-454		Up	48
				miR-19a	PTEN and CCND1	Up	48
				miR135a	APC	Up	48
				miR-137		Up	48
				miR-31		Up	48
				miR-129-3p	CDK6	Up	48
				miR-224		Up	48
				miR-10b	NF1, HOXA3 and HOXD10	Up	48
				miR-148a		Up	48
				miR-18a		Up	48
				miR-452		Up	48
				miR-598		Up	48
				miR-196b	HOXB8	Up	48
				miR-93		Up	48
				miR-20a	CCND1 and E2F1	Up	48
				miR-19b		Up	48
				miR-484		Up	48
				miR-192		Up	48
		High-grade VS low-grade glioma miRNA profiling	MicroRNA arrays	miR-10b-5p	CRLF3, Bim, TFAP2c, p16, RhoC and uPAR	Up	49
				miR-135b-5p		Up	49
				miR-196a-5p		Up	49
				miR-196b-5p		Up	49
				miR1247-5p	Wnt/β-catenin	Up	49
				miR320a	SND1 and β-catenin	Up	49
				miR-452a		Up	49
				miR-378b		Down	49
				miR-1305		Down	49
	NS	Study of the progression of JMML in *PTPN11* NS patients	RT-qPCR	miR-223		Up	50
				miR-15a		Up	50
**lncRNA**	NF1	Optic gliomas and PNFs tumor development	RT-qPCR	ARNIL (polymorphism rs2151280)	Stabilization of PRC1-2 repressive complexes in *CDKN2A/B*	Up	52
	NS	Molecular diagnosis by detection of DNM3OS deletion	oaCGH	DNM3OS	NGF	Mutated	46

Supplementary Table S3. ncRNA biomarkers in RASopathies.

Component	PTMs	Description	Activity	Reference
NF1	Phosphorylation	Modulates interaction with 14-3-3 protein	Affects GAP activity towards Ras	65
NF1	Phosphorylation	Residues S2808, S2811	Influences neurofibromin function in RAS signaling	64
NF1	Phosphorylation	Potential residues sites: S620, S859, S1815, S2578, S2741	Predicted influence on neurofibromin activity	62
NF1	Methylation	Conserved residues sites in Mus musculus: R366, R664, R1132, R1134, R1139, R1278, R1418	Modulates neurofibromin activity	66
HRAS/NRAS	Palmitoylation	Sites: C181, C184	Affects subcellular localization and activity of RAS	59
RAS	Ubiquitination	Regulation of degradation	Regulates protein levels	55;56
HRAS	Phosphorylation	Sites: T144, T148 by GSK3β	Leads to polyubiquitination and degradation	57
RAS	Methylation	Lysine residues: K5, K147	Modulates activity, impacting cell growth and proliferation	58
BRAF	Phosphorylation	Sites: S338, Y341	Aberrant activation in NS	69
c-RAF (RAF1)	Phosphorylation	Site: S259	Prevents inactivation in NS	70
RAF	Phosphorylation	Site: S621	Contributes to pathogenesis in NS	69
RAF	Ubiquitination	Affects stability	Contributes to abnormal signaling	71
MEK2	Phosphorylation	Site: T286	Affects localization and activity in RASopathies	1
MEK	Acetylation	Influence on activity	Affected by mutations in regulatory genes	79
ERK	Phosphorylation	Sites: T202, Y204	Affects activation in NS and RASopathies	72
ERK2	Phosphorylation	Sites: T185, Y187	Contributes to abnormal signaling in RASopathies	1
ERK	Acetylation	Affects activity	Influenced by mutations in ERK genes in RASopathies	1
ERK	Ubiquitination	Regulates degradation	Contributes to aberrant signaling	73
KRAS	O-GlcNAcylation	Addition of N-acetylglucosamine to serine/threonine residues	Modulates signaling activity	57
RAS	S-nitrosylation	Addition of nitroso group (NO) to cysteine residues	Alters protein-protein interactions	77
RAF	Cross-talk Ubiquitination and SUMOylation	Interplay between ubiquitination and SUMOylation regulates stability and subcellular localization	Sustains signaling	137
ERK	ADP-ribosylation	Addition of ADP-ribose to proteins	Regulates enzymatic activity and substrate interaction	78
RAS Pathway	Serine 181 in KRAS4B	Phosphorylation site	Regulation of KRAS activity	75
	Threonine 144/148 in HRAS	Phosphorylation site by GSK3β	Degradation of HRAS	57
	RAS Ubiquitination	Post-translational modification	Regulation protein degradation	55;56
	SUMOylation of RAS at Lys42	Post-translational modification	Activation of RAS signaling pathways	54;75
	Methylation of Lys5 and Lys147 in RAS	Post-translational modification	Modulation of binding affinity and activity of RAS	58
mTOR	Phosphorylation	Found in *PTPN11*-caused glioneuronal neoplasm	Activation of PI3/AKT/mTOR pathway	16
pAKT, pS6RP	Phosphorylation	Found in a NSML skin fibroblasts	Activation of PI3/AKT/mTOR pathway	80
AKT, S6RP, mTOR	Phosphorylation	Found in NF1 MPNSTs	Activation of PI3/AKT/mTOR pathway	82
NF1	Phosphorylation	By PKA	Impairment of GAP activity and dysregulation of RAS/MAPK pathway	62
STAT5	Dephopshorylation	By SHP2	Attenuation of its activity by inhibiting dimerization	97
JAK	Dephopshorylation	By SHP2	Attenuation of its activity by inhibiting dimerization	97

Supplementary Table S4. PTMs in RASopathies.
